# ﻿Revision of two species of *Sinopotamon* Bott, 1967 (Crustacea, Brachyura, Potamidae) endemic to China: a new combination and a new synonym

**DOI:** 10.3897/zookeys.1112.85278

**Published:** 2022-07-14

**Authors:** Ruxiao Wang, Da Pan, Hui Chen, Binqing Zhu, Hongying Sun

**Affiliations:** 1 Jiangsu Key Laboratory for Biodiversity and Biotechnology, College of Life Sciences, Nanjing Normal University, 1 Wenyuan Rd, Nanjing 210023, China Nanjing Normal University Nanjing China; 2 Nanjing Institute of Environmental Sciences, Ministry of Ecology and Environment, Nanjing 210042, China Nanjing Institute of Environmental Sciences, Ministry of Ecology and Environment Nanjing China; 3 Research Station for Ecological Environment of Wuyi Mountains/Biodiversity Comprehensive Observation Station for Wuyi Mountains/State Environmental Protection Key Laboratory on Biosafety, Nanping, 353000, China Research Station for Ecological Environment of Wuyi Mountains/Biodiversity Comprehensive Observation Station for Wuyi Mountains/State Environmental Protection Key Laboratory on Biosafety Nanping China

**Keywords:** 16S rDNA, *Huananpotamonkoatenense* comb. nov., *Sinopotamonwuyiensis*, systematics, taxonomy, Wuyishan National Park

## Abstract

The systematics of two problematic potamid species, *Sinopotamonkoatenense* (Rathbun, 1904) and *Sinopotamonwuyiensis* Li, Lin, Cheng & Tang, 1985, both originally described from the Wuyi Mountains are resolved in this study. *Sinopotamonkoatenense* is transferred to the genus *Huananpotamon* Dai & Ng, 1994, as the new combination *Huananpotamonkoatenense***comb. nov.** The new combination differs from its congeners in the form of the carapace, male pleon, male first gonopod, and vulvae. Phylogenetic analyses based on mitochondrial 16S rDNA sequences support the identification of *Huananpotamonkoatenense***comb. nov.** and a redescription is also provided. In addition, *S.wuyiensis* is confirmed as a junior synonym of *Sinopotamonfukienense* Dai & Chen, 1979 based on morphological similarities and phylogenetic lineages.

## ﻿Introduction

Guadun, also spelled Koaten or Kuatun, is located in Tongmu Village in the Wuyi Mountains, which has been under administration of the Wuyishan National Park since 2021; it is a well-known type locality for several herpetofauna species, e.g., *Boulenophryskuatunensis* (Pope, 1929), *Opisthotropiskuatunensis* Pope, 1928. As early as 1873, Father Armand David collected a female freshwater crab specimen from Guadun, and preserved it at the Muséum national d’Histoire naturelle, Paris (MNHN). Rathbun (1904) recognized the specimen as a new species, Potamon (Potamon) koatenensis Rathbun, 1904. With the establishment of the genus *Sinopotamon* Bott, 1967, Potamon (P.) koatenensis was then transferred to *Sinopotamon*, but had been regarded as a synonym of *Sinopotamondenticulatum* (A. Milne-Edwards, 1853) (Bott 1970). Although Ng et al. (2008) considered *Sinopotamonkoatenense* as a valid species, Chu et al. (2018) synonymized *S.koatenense* with *Sinopotamonfukienense* Dai & Chen, 1979. To date, the taxonomic status and validity of *S.koatenense* has been debated for more than a century.

*Sinopotamonwuyiensis* Li, Lin, Cheng & Tang, 1985, was described from the Sangang, Tongmu Village in the Wuyi Mountains and has not been reported since its original description. Notably, except for Chu et al. (2018), neither Dai (1999) nor Ng et al. (2008) had listed the species in their species checklists. Consequently, the validity of *S.wuyiensis* has also been unconfirmed for nearly 40 years.

**Figure 1.**
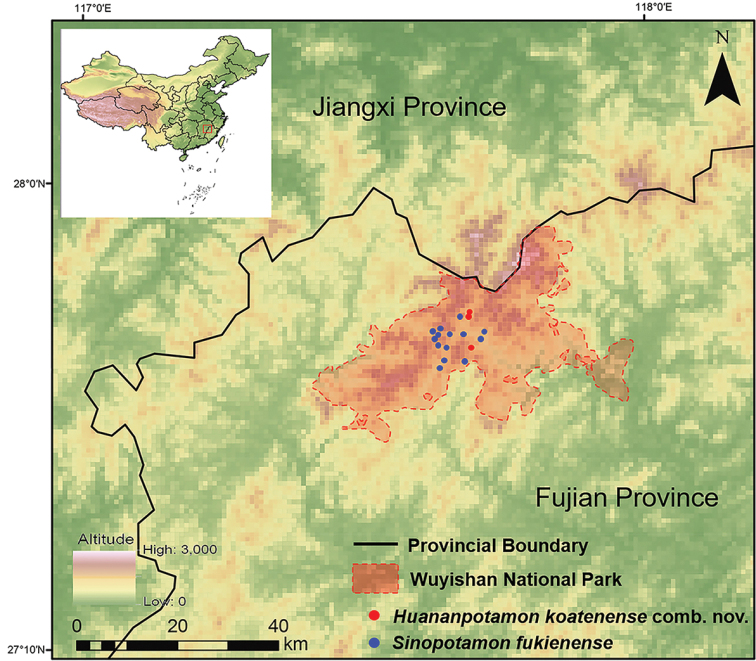
Localities of the sampling sites for *Huananpotamonkoatenense* and *Sinopotamonfukienense* in the Wuyishan National Park, Fujian.

Recently, we conducted comprehensive surveys of freshwater crabs in the Wuyishan National Park and collected several specimens from the type localities of the abovementioned species (Fig. 1). Our morphological comparisons with the holotype of *S.koatenense* indicate that it was erroneously placed in *Sinopotamon*, and should be transferred to *Huananpotamon* Dai & Ng, 1994, as *Huananpotamonkoatenense* comb. nov. Molecular phylogenetic analyses and detailed morphological comparisons with other *Huananpotamon* species indicate that the new combination as a species of *Huananpotamon*. The new combination is redescribed in the present study. In addition, *S.wuyiensis* is revised herein, suggesting that it is in fact a junior synonym of *S.fukienense*.

## ﻿Material and methods

We conducted three comprehensive surveys of freshwater crabs at the Wuyishan National Park in March, November, and December of 2021. Specimens were collected by hand, preserved in 95% ethanol, and deposited in the freshwater crab collection of Jiangsu Key Laboratory for Biodiversity and Biotechnology, College of Life Sciences, Nanjing Normal University (**NNU**), Nanjing, China. Measurements, in millimeters, are of carapace width and length, respectively. The abbreviations used in the text and figures are as follows: **G1**, male first gonopod; **G2**, male second gonopod; and a.s.l., above sea level. The terminology used here follows that of Dai (1999) and Davie et al. (2015).

Shih et al. (2016) proposed that *Sinopotamon* Bott, 1967 be split into two genera, *Sinopotamon* s. str. and *Longpotamon* Shih, Huang & Ng, 2016. The authors, however, indicate that *Sinopotamon* s. str. and *Longpotamon* each comprise two lineages based on extensive taxonomic sampling and comprehensive multi-locus phylogeny, and that another two new genera should be established. Therefore, until such taxonomic actions are made, we follow the classical taxonomic system proposed by Bott (1967) for *Sinopotamon*.

Total genomic DNA was extracted from gill tissue using the Trelief^TM^ Animal Genomic DNA kit (Tsingke). 16S rDNA sequences were amplified using the primers 1471 and 1472 (Crandall and Fitzpatrick 1996). The protocol as follows: initial denaturation for 3 min at 95 °C, 35 cycles of denaturation for 15 s at 95 °C, annealing for 15 s at 48 °C, extension for 15 s at 72 °C, and final extension for 5 min at 72 °C. PCR products were sequenced on an ABI 3730 automatic sequencer. Newly generated sequences were deposited in GenBank (Table 1).

**Table 1.** The 16S rDNA sequences used in the phylogenetic analysis.

Species	Locality	Accession No.	Reference
Ingroup taxa			
*Apotamonauteshainanensisbawanglingensis*	Haikou, Hainan	MN737137	Pan et al. 2022
*Apotamonauteshainanensishainanensis*	Lean, Hainan	AB428459	Shih et al. 2009
*Bottapotamonlingchuanense*	Gongcheng, Guangxi	NC049011	Pan et al. 2022
*Candidiopotamonokinawense*	Ryukyus, Okinawa	AB208627	Shih et al. 2006
*Chinapotamondepressum*	Mashan, Guangxi	MZ350918	Pan et al. 2022
*Geothelphusadehaani*	Chiba, Japan	AB290630	Yeo et al. 2007
*Geothelphusaalbogilva*	Pingdong, Taiwan	MZ350921	Pan et al. 2022
*Hainanpotamondaiae*	Lingshu, Hainan	MZ350922	Pan et al. 2022
*Hainanpotamonfuchengense*	Haikou, Hainan	AB428461	Shih et al. 2009
*Huananpotamonangulatum*	Fuzhou, Fujian	AB428454	Shih et al. 2009
*Huananpotamonchongrenense*	Chongren, Jiangxi	ON454341	This study
*Huananpotamonguixiense*	Guixi, Jiangxi	ON505056	This study
*Huananpotamonkoatenense*	Wuyishan, Fujian	ON505057	This study
*Huananpotamonkoatenense*	Wuyishan, Fujian	ON505058	This study
*Huananpotamonlichuanense*	Lichuan, Jiangxi	MN737141	Pan et al. 2022
*Huananpotamonlini*	Songxi, Fujian	ON454342	This study
*Huananpotamonmedium*	Nancheng, Jiangxi	ON454342	This study
*Huananpotamonsheni*	Shaowu, Fujian	ON505059	This study
*Huananpotamonyiyangense*	Yiyang, Jiangxi	ON505060	This study
*Minpotamon* sp.	Longhai, Fujian	ON454347	This study
*Minpotamonkityang*	Jieyang, Guangdong	MN253481	Mao and Huang 2020
*Minpotamonnasicum*	Longhai, Fujian	AB428450	Shih et al. 2009
*Nanhaipotamonhongkongense*	Hong Kong, China	AB212869	Shih et al. 2005
*Nanhaipotamonnanriense*	Putian, Fujian	AB212868	Shih et al. 2005
*Nanhaipotamonpinghense*	Heping, Guangdong	AB433553	Shih et al. 2011
*Nanhaipotamonpingyuanense*	Pingyuan,Guangdong	AB265237	Shih et al. 2006
*Nanhaipotamonwupingense*	Wuping, Fujian	AB470496	Shih et al. 2011
*Nanhaipotamonyongchuense*	Yongchun, Fujian	AB433546	Shih et al. 2011
*Neotiwaripotamonjianfengense*	Changjiang, Hainan	MZ350933	Pan et al. 2022
*Neotiwaripotamonwhiteheadi*	Qiongzhong, Hainan	MZ350934	Pan et al. 2022
*Qianguimonelongatum*	Guangxi, China	MG709240	Huang 2018
*Sinolapotamonpatellifer*	Lingchuan, Guangxi	MZ350948	Pan et al. 2022
Outgroup taxa			
*Acantiapotamoninflatum*	Xuanen, Hubei	MZ350902	Pan et al. 2022
*Aiyunamonlushuiense*	Lushui, Yunnan	MZ350919	Pan et al. 2022
*Indochinamonjianchuanense*	Jianchuan, Yunnan	MZ350928	Pan et al. 2022
*Latopotamonobtortum*	Shuicheng, Guizhou	MZ350930	Pan et al. 2022
*Parapotamonoidesendymion*	Kunming, Yunnan	MZ350935	Pan et al. 2022
*Potamiscusyiwuensis*	Yunnan, China	AB428476	Shih et al. 2009
*Sinopotamonfukienense*	Wuyishan, Fujian	ON505061	This study
*Sinopotamonfukienense*	Shaowu, Fujian	KT586120	Ji et al. 2016
*Tenuilapotamonjoshuiense*	Leishan, Guizhou	MZ350951	Pan et al. 2022
*Tenuilapotamonlatilumlatilum*	Xianfeng, Hubei	MN737132	Pan et al. 2022
*Vadosapotamonsheni*	Rongjing, Sichuan	MZ350958	Pan et al. 2022

A total of 43 sequences from the ingroup and outgroup taxa were used in the phylogenetic analyses, including 33 downloaded sequences (Table 1). Pan et al. (2022) showed that the genus *Huananpotamon* nested within the “South China-adjacent Islands” clade; therefore, closely related genera from the same clade, in particular the taxa of closely related genera from Fujian, Guangdong, and Jiangxi, and some others from adjacent islands, were included for the present phylogenetic analyses (Table 1). For the genus *Huananpotamon*, eight species from the adjacent area of the Wuyishan National Park were included, of which four species are morphologically most similar to *H.koatenense* (Table 1; Fig. 9). Ten species belonging to the closely related “Central China” and “Indochina-Southwest China” clades (Pan et al. 2022) were used as outgroups. Among them, two specimens of *S.fukienense*, collected from Guadun (Koaten) and Shaowu of Fujian Province were also included in our phylogenetic analyses (Table 1).

All sequences were aligned using MAFFT 7.487 (Katoh and Standley 2013) based on the G-INS-I method. The best-fit model was selected using jModelTest 2.1.10 (Darriba et al. 2012). Maximum likelihood (ML) analysis was implemented in IQ-TREE 1.6.12 (Nguyen et al. 2015) using the ultrafast bootstrapping approach (Minh et al. 2013) with 1000 replicates. MrBayes 3.2.7a (Ronquist et al. 2012) was used to perform Bayesian inference (BI) analysis. Two independent runs were carried out. In each run, four MCMC chains were run for 30 million generations, with every 100 generations being sampled. Convergence of two runs was checked using Tracer 1.6 (Rambaut et al. 2014). All effective sampling sizes (ESS) values were more than 200. The first 25% of samples from the MCMC chain were discarded as burn-in. The pairwise estimates of Kimura 2-parameter (K2P) distances (Kimura 1980) were calculated using MEGA X (Kumar et al. 2018).

## ﻿Results

### ﻿Taxonomy

**Family Potamidae Ortmann, 1896**

**Subfamily Potamiscinae Bott, 1970**

#### Genus *Sinopotamon* Bott, 1967

##### ﻿ Sinopotamon fukienense

Taxon classificationAnimaliaDecapodaPotamidae

Dai & Chen, 1979

3FFBDEF4-9D18-5559-B97E-C658B048620E

Figs 2 , 3

Sinopotamon fukienense Dai & Chen, 1979: 247, fig. 2(1)–(3); Dai 1999: 259, pl. 17 (7), fig. 136.
Sinopotamon wuyiensis Li, Lin, Cheng & Tang, 1985: 144, figs 1–6 [synonymy].

###### Material examined.

2 males, 23.2 × 29.0 mm, NNU11B-211121SF1; 22.0 × 27.6 mm, NNU 11B-211121SF2, Guadun, Wuyishan National Park, Fujian, coll. Caixin Liu, Kangqin Zhang, Ruxiao Wang & Hongying Sun, 21 November 2021. 3 female, 17.5 × 22.3 mm, NNU 11B-211121SF3; 19.5 × 25.4 mm, NNU 11B-211121SF4; 20.5 × 26.1 mm, NNU 11B-211121SF5, same data as above. 1 male, 22.5 × 28.1 mm, NNU 11B-211217SF6, Guadun, Wuyishan National Park, Fujian, coll. Hui Chen, Yunlong Sun, Ruxiao Wang & Hongying Sun, 17 December 2021. 2 males, 17.9 × 22.2 mm, NNU 11B-211217SF7; 17.6 × 22.3 mm, NNU 11B-211217SF8, Tongmuguan, Wuyishan National Park, Fujian, same data as above. 1 female, 16.8 × 21.0 mm, NNU 11B-211217SF9, same data as above. 4 females, 17.9 × 23.7 mm, NNU 11B-211121SF10, Dazhulan, Wuyishan National Park, Fujian, coll. Caixin Liu, Kangqin Zhang, Ruxiao Wang & Hongying Sun, 21 November 2021. 3 females, 22.3 × 28.0 mm, NNU 11B-211215SF11; 17.0 × 21.2 mm, NNU 11B-211215SF12; 26.3 × 33.4 mm, NNU 11B-211215SF13, same data as above, 15 December 2021. 2 males, 22.5 × 29.0 mm, NNU 11B-211219SF14; 21.3 × 27.2 mm, NNU 11B-211219SF15, Longdu, Wuyishan National Park, Fujian, coll. Hui Chen, Yunlong Sun, Ruxiao Wang & Hongying Sun, 19 December 2021. 1 female, 21.8 × 27.7 mm, NNU 11B-211219SF16, same data as above. 1 male, 19.6 × 24.9 mm, NNU 11B-211218SF17, Qiaoxia, Wuyishan National Park, Fujian, coll. Hui Chen, Yunlong Sun, Ruxiao Wang & Hongying Sun, 18 December 2021. 1 female, 17.9 × 22.8 mm, NNU 11B-211218SF18, same data as above. 1 female, 35.5 × 43.4 mm, NNU 11B-211218SF19, Jiangdun, Wuyishan National Park, Fujian, same data as above.

###### Distribution and habitat.

Jiangxi Province, and Minjiang basin in Fujian Province. The crabs were found under rocks in mountain streams.

###### Remarks.

**Figure 2.**
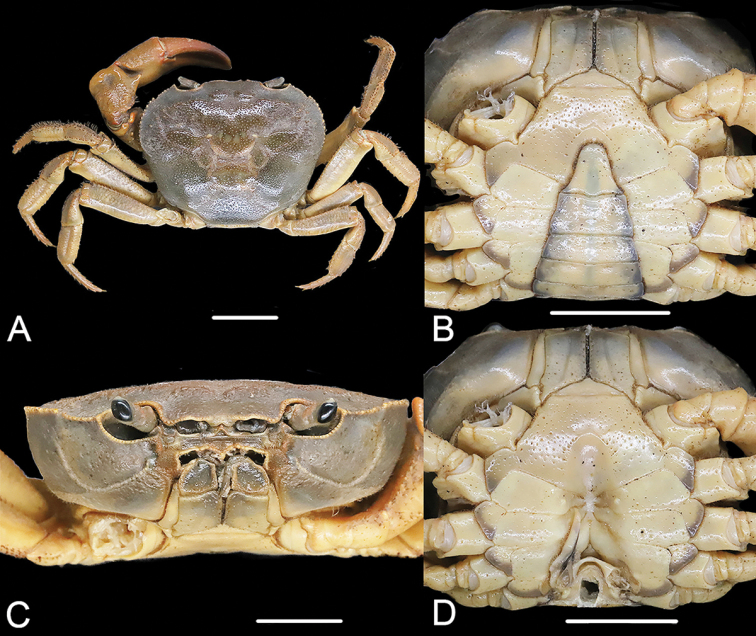
*Sinopotamonfukienense*, male, 23.2 × 29.0 mm, NNU11B-211121SF1 **A** dorsal overall view ventral **B** view of anterior thoracic sternum and pleon **C** frontal view **D** ventral view of sterno-pleonal cavity with right G1 in situ (left G1 removed). Scale bars: 1.0 cm.

**Figure 3.**
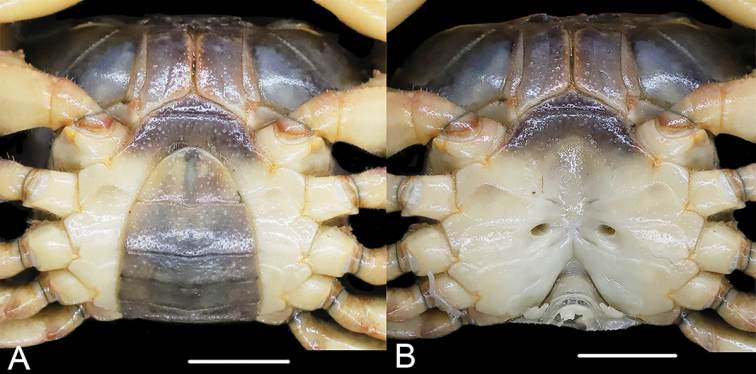
*Sinopotamonfukienense*, female, 22.5 × 29.0 mm, NNU 11B-211219SF14 **A** ventral view of pleon **B** ventral view showing female vulvae. Scale bars: 1.0 cm.

*Sinopotamonwuyiensis* was considered a new species based on its distinct morphological characters in both male and female specimens, especially in the male specimen having a pair of vulvae on sternite 6, which is not seen in any known species of *Sinopotamon*. Unfortunately, the type material of *S.wuyiensis*, which was originally deposited in Fujian Research Institute of Parasite Disease, Fuzhou, Fujian Province, is now lost (Youzhu Cheng, pers. comm.). Therefore, to investigate the status and validity of *S.wuyiensis*, we conducted comprehensive collections in Tongmu Village but found no record of *S.wuyiensis* in the area. Instead, we collected 21 new specimens from 14 localities within the area (Fig. 1), all of which were identified as *S.fukienense* by morphological comparison (Figs 2, 3). According to the original description of *S.wuyiensis*, apart from the unique male sternite 6, *S.wuyiensis* is identical with *S.fukienense* in most characters, including outer margin of external orbital angle lined with 5–7 granules (Fig. 2A; cf. Dai and Chen 1979; Li et al. 1985; Dai 1999); anterolateral margin ~ 2× as long as external orbital outer margin (Fig. 2A; cf. Dai and Chen 1979; Li et al. 1985; Dai 1999); G1 terminal segment tapering, tip directed outwards (Fig. 2D; cf. Dai and Chen 1979: fig. 2(1), (2); Li et al. 1985: figs 1, 2; Dai 1999: fig. 136(3), (4)), not reaching suture sternites 5/6 (Fig. 2D; cf. Dai and Chen 1979: fig. 2(1), (2); Li et al. 1985: figs 1, 2; Dai 1999: fig. 136(3), (4)), subterminal segment to terminal segment ratio ~ 2.5 (Fig. 2D; cf. Dai and Chen 1979; Li et al. 1985; Dai 1999); female pleon subovate, vulvae ovate (Fig. 3; cf. Dai and Chen 1979; Li et al. 1985: figs 5, 6; Dai 1999: fig. 136(7), (8)). Therefore, it is most likely that the holotype of *S.wuyiensis* is a rare gynandromorphic specimen (Youzhu Cheng, pers. comm.), rather than a representative of a new species. Therefore, *S.wuyiensis* is herein synonymized with *S.fukienense*.

#### Genus *Huananpotamon* Dai & Ng, 1994

##### ﻿ Huananpotamon koatenense

Taxon classificationAnimaliaDecapodaPotamidae

(Rathbun, 1904) comb. nov.

CB6DC1E5-0706-5F8E-9E4B-907940E3BFE5

**Figure 4.**
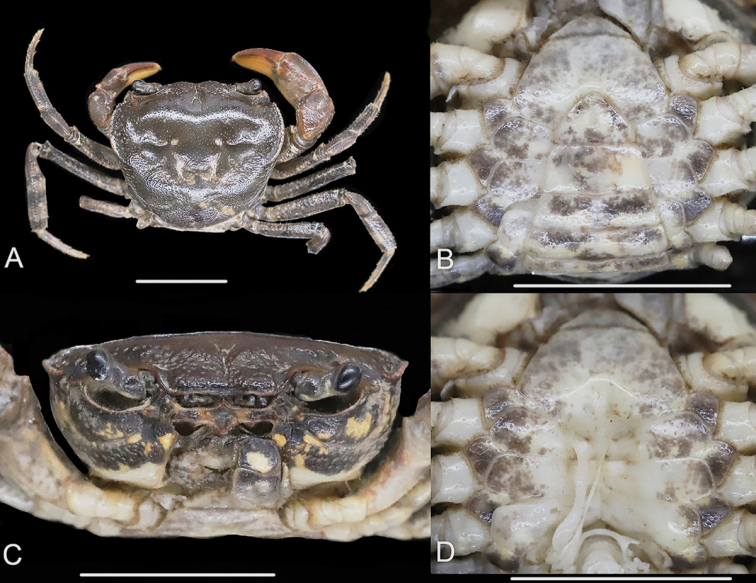
*Huananpotamonkoatenense*, male, 15.8 × 19.2 mm NNU 16C-211220HK1 **A** dorsal view **B** ventral view **C** frontal view **D** ventral view showing sterno-pleonal cavity with right G1 in situ. Scale bars: 1.0 cm.

**Figure 5.**
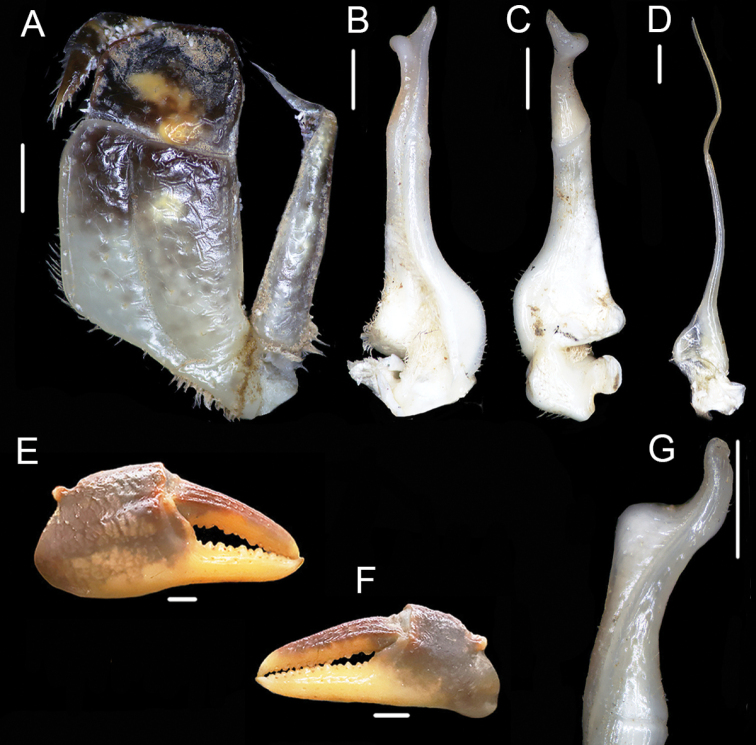
*Huananpotamonkoatenense*, male, 15.8 × 19.2 mm, NNU 16C-211220HK1 **A** left third maxilliped **B** left G1 (dorsal view) **C** left G1 (ventral view) **D** left G2**E** male right cheliped **F** male left cheliped **G** left G1 terminal segment (lateral view). Scale bars: 1.0 mm.

**Figure 6.**
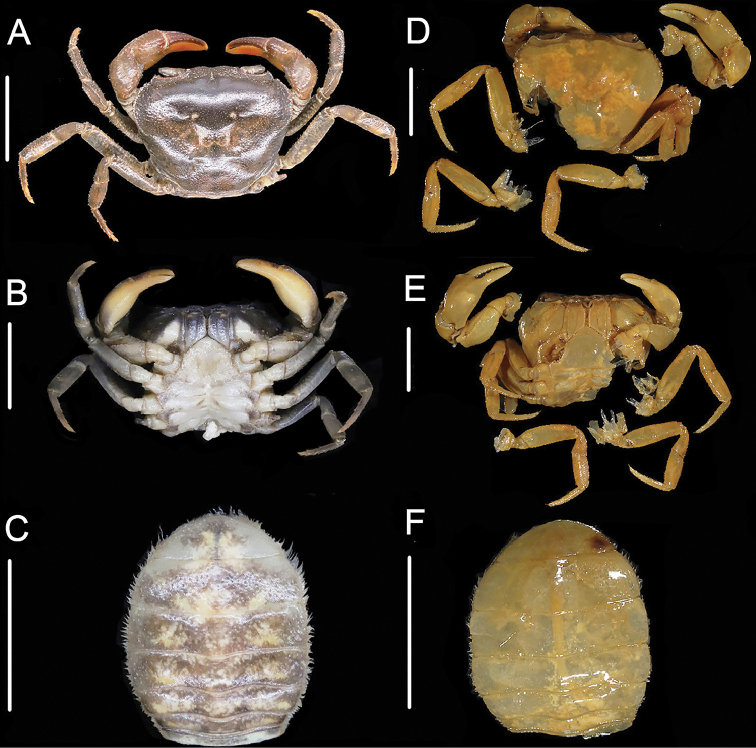
Female specimens compared in this study: *Huananpotamonkoatenense*, 19.7 × 25.4 mm, NNU 16C-211220HK2 **A** dorsal view **B** thoracic sternum and vulvae **C** pleon. *H.koatenense*, holotype, 18.4 × 23.4 mm, MNHN-IU-2014-23011 **D** dorsal view **E** thoracic sternum and vulvae **F** pleon. Scale bars: 1.0 cm.

**Figure 7.**
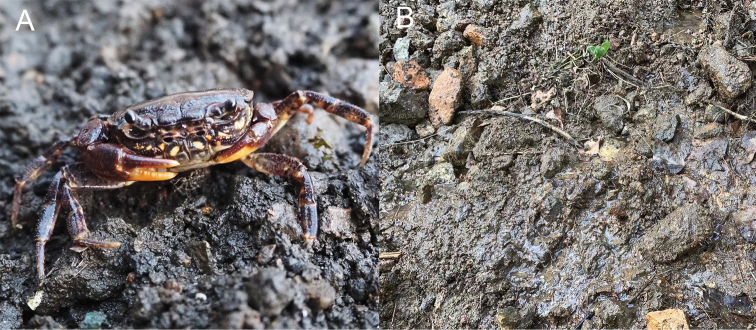
*Huananpotamonkoatenense***A** color in life **B** natural habitat. Photographs by Hongying Sun, 20 December 2021, Tongmu Village, Wuyishan National Park, Fujian Province, China.

**Figure 8.**
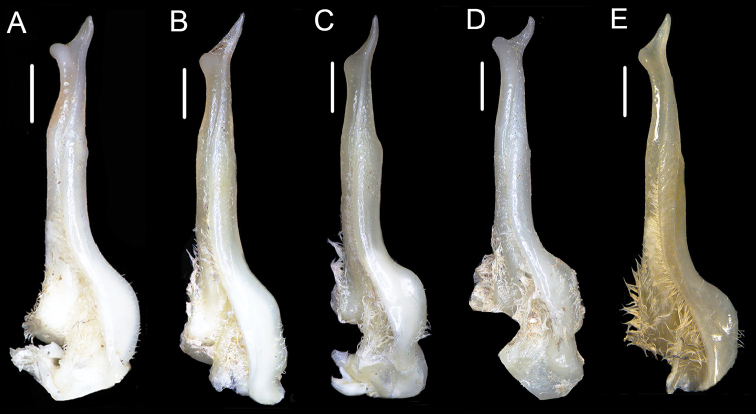
The ventral view of G1**A***Huananpotamonkoatenense*, 15.8 × 19.2 mm, NNU 16C-211220HK1 **B***Huananpotamonangulatum*, male, 14.3 × 18.9 mm, NNU 11B-21413HA1 **C***Huananpotamonlichuanense*, male, 12.0 × 14.4 mm, NNU 11B-21320HL1 **D***Huananpotamonlini*, male, 16.5 × 18.8 mm, NNU 11B-211225HL1 **E***Huananpotamonyiyangense*, male, 13.7 × 16.3 mm, NNU 11B-21415HY1. Scale bars: 1.0 mm.

Figs 4 , 5 , 6 , 7A , 8A

Patamon (Potamon) koatenensis Rathbun, 1904: 308–309, pl. 13, fig. 3.
Sinopotamon koatenense (Rathbun, 1904): Ng et al. 2008: 166.

###### Type material.

Potamon (P.) koatenensis, ***holotype***, 1 female, 18.4 × 23.4 mm, MNHN-IU-2014-23011 (citation: http://coldb.mnhn.fr/catalognumber/mnhn/iu/2014-23011), 1200 m a.s.l., Koaten, Western Fujian Province, China, coll. Armand David, October 1873.

###### Other material.

1 male 15.8 × 19.2 mm, NNU 16C-211220HK1, 27°42.40'N, 117°41.47'E, 890 m a.s.l., Guwangkeng, Tongmu Village, Wuyishan National Park, Fujian Province, China, coll. Hui Chen, Ruxiao Wang, Yunlong Sun & Hongying Sun, 20 December 2021. 2 females, 19.7 × 25.4 mm, NNU 16C-211220HK2; 14.9 × 18.8 mm, NNU 16C-211220HK3, same collection data as above. 2 females, 17.0 × 21.6 mm, NNU 16C-211220HK4; 14.3 × 17.6 mm, NNU 16C-211220HK5, 27°45.77'N, 117°41.23'E, 854 m a.s.l., Qiaoxia, Tongmu Village, Wuyishan National Park, Fujian Province, China, coll. Hui Chen, Ruxiao Wang, Yunlong Sun & Hongying Sun, 17 December 2021. 1 female, 13.6 × 16.8 mm, NNU 16C-211220HK6, 27°46.15'N, 117°41.28'E, 893 m a.s.l., Jiangdun, Tongmu Village, Wuyishan National Park, Fujian Province, China, coll. Hui Chen, Ruxiao Wang, Yunlong Sun & Hongying Sun, 18 December 2021.

###### Comparative material.

*Huananpotamonangulatum* (Dai, Chen, Song, Fan, Lin & Zeng, 1979), male, 14.3 × 18.9 mm, NNU 11B-21413HA1, Jianou, Fujian, 1 April 2021; *Huananpotamonlichuanense* Dai, Zhou & Peng, 1995, male, 12.0 × 14.4 mm, NNU 11B-21320HL1, Lichuan, Jiangxi, 20 March 2021; *Huananpotamonlini* Cheng & Li, 2008, male, 16.5 × 18.8 mm, NNU 11B-211225HL1, Songxi, Fujian, 25 December 2021; *Huananpotamonyiyangense* Dai, Zhou & Peng, 1995, male, 13.7 × 16.3 mm, NNU 11B-21415HY1, Yiyang, Jiangxi, 15 April 2021.

###### Diagnosis.

Carapace broader than long, dorsal surface slightly convex, finely pitted (Figs 4A, 6A, D); frontal margin distinctively bilobed, separated medially by shallow concavity (Figs 4A, 6A, D); epigastric cristae prominent, separated medially by distinct Y-shaped furrow (Figs 4A, 6A, D); postorbital cristae distinct, slightly rugose, confluent with epibranchial teeth (Figs 4A, 6A, D); anterolateral region convex, with rugose (Figs 4A, C, 6A, D). Third maxilliped exopod with flagellum (Figs 4A, 6A). G1 slender; tip part of terminal segment expanded, inner-distal angle prominent, outer-distal angle elongated, dagger-shaped (Figs 5B, G, 8A). G2 slender, terminal segment long (Fig. 5D). Vulvae without operculum, not reaching suture of sternites 5/6 anteriorly, closely spaced from one another, opening inwards (Fig. 6B).

###### Description.

Carapace broader than long, regions distinct, dorsal surface gently convex, finely pitted (Figs 4A, 6A, D). Frontal margin distinctively bilobed, divided into two broad lobes, separated by shallow concavity, margin of each lobe gently convex (Figs 4A, 6A, D). Epigastric cristae prominent, separated medially by distinct Y-shaped furrow extending to frontal region (Figs 4A, 6A, D); postorbital cristae distinct, slightly rugose, confluent with epibranchial teeth (Figs 4A, 6A, D). Cervical grooves distinct, deep (Figs 4A, 6A, D); H-shaped groove shallow but distinct (Figs 4A, 6A, D); anterolateral region convex with weak rugae; posterolateral surface smooth, with oblique striae; posterolateral margins converging posteriorly (Figs 4A, 6A, D). External orbital angle distinct, sharp, triangular, outer margin longer in length to inner margin, outer margin lined with small granules (Figs 4A, 6A, D). Epibranchial tooth granular, clearly demarcated from external orbital tooth by small gap (Figs 4A, 6A, D). Anterolateral margin convex, lined with 15–18 granules (Figs 4A, C, 6A, D). Orbits ovate, large; supraorbital, infraorbital margins cristate, lined with numerous inconspicuous granules (Fig. 4C). Epistome posterior margin with median lobe broadly triangular, lateral margins almost straight (Fig. 4C).

Ischium of third maxillipeds trapezoidal, length ~ 1.3× width, with distinct median oblique groove; merus subquadrate, length ~ 0.8× width (Figs 5A, 6B, E); exopod slender, reaching proximal one-third of merus length, with flagellum (Fig. 5A).

Chelipeds slightly asymmetrical (Figs 4A, 6A, D). Merus margins crenulated (Fig. 4A). Carpus surface wrinkled with sharp spine on inner margin, spinule at base (Fig. 4A). Major cheliped palm length ~ 1.4× height, surface rugose; fingers of major cheliped slightly curved, outer surface with rows of pits; dactylus 1.1× as long as palm length. Occlusal margin of both fingers lined with 11–15 irregular small teeth, forming small gape when fingers closed (Fig. 5E, F).

Ambulatory legs slender, surfaces and margins with scattered short setae (Figs 4A, 6A, D); second ambulatory leg longest, merus ~ 1.5× as long as dactylus (Figs 4A, 6A, D).

Thoracic sternum surface generally smooth, weakly pitted (Figs 4B, D, 6B, E); sternites 1, 2 fused, forming triangular structure, separated from sternite 3 by conspicuous suture; sternites 3, 4 completely fused without obvious median suture (Figs 4B, 6B, E). Male sterno-pleonal cavity reaching to imaginary line connecting median part of cheliped coxae (Fig. 4B, D). Male pleonal locking tubercle on median part of sternite 5 (Fig. 4D).

Male pleon triangular, surface smooth (Fig. 4B, D); somites 3–6 gradually decreasing in width; somite 6 width ~ 2.0× length; telson broad triangular, width ~ 1.6× length, apex rounded (Fig. 4B). Female pleon ovate; somite 6 width ~ 3.4× length; telson semicircular, width ~ 2.9× length (Fig. 6C, F).

G1 slender, bent dorsal-ward at proximal one-thirds (Fig. 5B, C); tip well exceeding pleonal locking tubercle in situ, reaching suture between thoracic sternites 4, 5 (Fig. 4D); subterminal segment length ~ 2.1× length of terminal segment; distal part of subterminal segment almost straight; terminal segment slender, inner margin slightly concave, outer margin straight, distally expanded, inner-distal angle high, rounded, outer-distal angle elongated, dagger-shaped (Fig. 5B, C, G); groove for G2 running mid-line of ventral surface (Fig. 5B). G2 slender, longer than G1, subterminal segment length ~ 1.7× length of terminal segment (Fig. 5D). Female vulvae on thoracic sternite 6, elongate-ovate, without distinct operculum, closely spaced from one another, opening inwards (Fig. 6B).

###### Live coloration.

Generally dark brown to purplish brown. The immovable finger of the chelipeds and surrounding areas are pale yellow (Fig. 7A).

###### Distribution and habitat.

Tongmu Village, Wuyishan National Park, Fujian Province, China. This species lives in moist mud burrows under rocks near the small hillstream, ca 900 m a.s.l. (Fig. 7B). *Bottapotamonengelhardti* (Bott, 1967) was found to be sympatric with *H.koatenense* and appears to occupy the shallower parts of the streams and hides under rocks.

###### Remarks.

Thanks to digitalization of the specimen, we are able to examine the photographs of the holotype of *S.koatenense* provided by MNHN (http://coldb.mnhn.fr/catalognumber/mnhn/iu/2014-23011). Potamon (P.) koatenensis can be distinguished from *Sinopotamon* by several characters: small sized (versus large sized in *Sinopotamon*; cf. Bott 1967; Dai 1999), external orbital angle acutely triangular (Fig. 6D) (versus external orbital angle broadly triangular in *Sinopotamon*; cf. Bott 1967; Dai 1999), maxilliped 3 ischium relatively broad (Fig. 6E) (versus maxilliped 3 ischium relatively narrow in *Sinopotamon*; cf. Bott 1967; Dai 1999; fig. 139(1)), ambulatory legs slender (Fig. 6D, E) (versus ambulatory legs stout in *Sinopotamon*; cf. Bott 1967; Dai 1999), male sterno-pleonal cavity relatively narrow (Fig. 6E) (versus male sterno-pleonal cavity relatively broad in *Sinopotamon*; cf. Bott 1967; Dai 1999), female pleon broadly ovate (Fig. 6F) (versus female pleon ovate in *Sinopotamon*; cf. Bott 1967; Dai 1999), vulvae relatively small and narrow (Fig. 6E) (versus vulvae relatively large and wide in *Sinopotamon*; cf. Bott 1967; Dai 1999). In contrast, the holotype of *S.koatenense* conforms well to the genus diagnosis for *Huananpotamon*: small sized (cf. Dai and Ng 1994; Dai 1999), postorbital region narrow (Fig. 6A, D; cf. Dai and Ng 1994; Dai 1999: pl. 7, fig. 7), external orbital angle acutely triangular (Fig. 6A, D; cf. Dai and Ng 1994; Dai 1999: pl. 7, fig. 7), maxilliped 3 ischium relatively broad (Fig. 6A, D; cf. Dai and Ng 1994: fig. 1; Dai 1999: fig. 66(1)), ambulatory legs slender (Fig. 6A, B, D, E; cf. Dai and Ng 1994; Dai 1999: pl. 7, fig. 7), male sterno-pleonal cavity relatively narrow (Fig. 6B, E; cf. Dai and Ng 1994: fig. 1; Dai 1999: fig. 66(8)), female pleon broadly ovate (Fig. 6C, F; cf. Dai and Ng 1994: fig. 1; Dai 1999: fig. 66(7)), vulvae small, ovate, not reaching the sutures of sternites 5/6 (Fig. 6B, E; cf. Dai and Ng 1994: fig. 1; Dai 1999: fig. 66(8)).

*Huananpotamonkoatenense* is close to four other *Huananpotamon* species, *H.angulatum* (Dai, Chen, Song, Fan, Lin & Zeng, 1979), *H.lichuanense* Dai, Zhou & Peng, 1995, *H.lini* Cheng & Li, and *H.yiyangense* Dai, Zhou & Peng, 1995 in having slender G1 terminal segment, and elongated outer-distal angle of G1 terminal segment (Fig. 8). However, the G1 terminal segment of *H.koatenense* can be distinguished from its congeners by the following characters: inner margin of G1 terminal segment slightly concave (Fig. 8A) (versus inner margin of terminal segment relatively straight in *H.lichuanense* and *H.lini*; cf. Dai et al. 1995: fig. 3; Cheng et al. 2008: fig. 1; Fig. 8C, D), G1 terminal segment with inner-distal angle relatively higher, globular in shape (Fig. 8A) (versus terminal segment with inner-distal angle relatively lower, somewhat blunt in *H.angulatum*, *H.lichuanense*, and *H.yiyangense*; cf. Dai and Ng 1994: fig. 1; Dai et al. 1995: figs 1, 3; Fig. 8B–C, E), G1 terminal segment with outer-distal angle relatively short (Fig. 8A) (versus terminal segment outer-distal angle relatively elongated in *H.angulatum*, *H.lichuanense*, and *H.yiyangense*; cf. Dai and Ng 1994: fig. 1; Dai et al. 1995: figs 1, 3; Fig. 8B–C, E). Other differences existing in the external orbital teeth, epibranchial teeth, and vulva are listed in Table 2. In this study, only one male specimen was collected, so we did not have enough specimens to analyze intraspecific variations and this matter may require further investigation.

**Table 2.** Morphological differences among *Huananpotamonkoatenense*, and the other four species of *Huananpotamon*, *H.chongrenense*, *H.lini*, *H.medium*, *H.obtusum*, and *H.ruijinense*.

Character	External orbital tooth	Epibranchial tooth	Cleft between external orbital and epibranchial teeth	Vulva
*H.koatenense*	Sharp (Fig. 4A)	Granular (Fig. 4A)	Shallow (Fig. 4A)	Ovate (Fig. 6B)
*H.angulatum* (cf. Dai & Ng, 1994: fig. 1)	Sharp	Granular	Shallow	Ovate
*H.lichuanense* (cf. Dai et al., 1995: fig. 3)	Acutely sharp	Granular	Shallow	Widely ovate
*H.lini* (cf. Cheng et al., 2008: fig. 1)	Acutely sharp	Rounded	Deep, U-shaped	Widely ovate
*H.yiyangense* (cf. Dai et al., 1995: fig. 1)	Acutely sharp	Rounded	Shallow	Ovate

### ﻿Phylogenetic analyses

**Figure 9.**
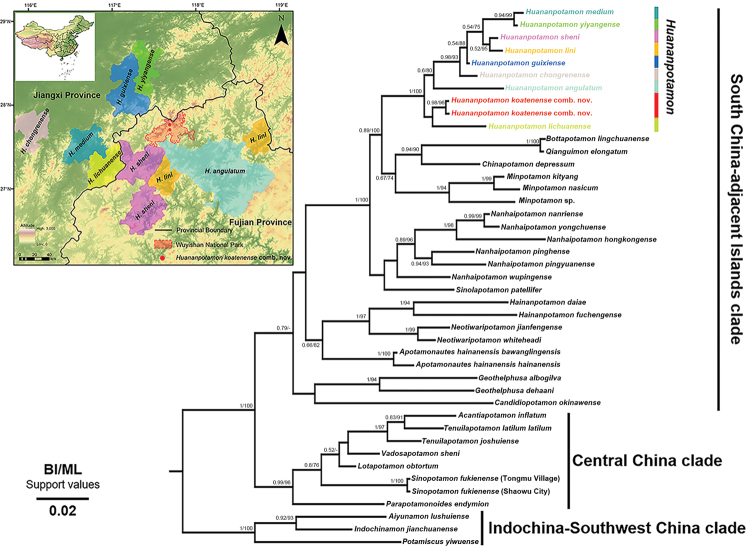
A Bayesian inference tree based on the 16S rDNA sequence. Values at the nodes represent bootstrap (BS) and posterior probability (BPP) values for ML and BI, respectively. Only values higher than 70/0.5 (BS/BPP) are shown. Distribution areas of eight *Huananpotamon* species are shown in the upper left map using the tree colors.

A total of 41 species from 21 genera were included in the present phylogenetic analyses. The phylogenetic trees were reconstructed using BI and ML and yielded similar phylogenetic topologies with some minor differences in the terminal lineages (Fig. 9). The phylogenetic relationship showed that the genus *Huananpotamon* is monophyletic and belongs to the “South China-adjacent Islands” clade (Pan et al. 2022) (Fig. 9). *Huananpotamonkoatenense* is genetically closest to *H.lichuanense* in the phylogenetic tree. Except for *H.angulatum*, the interspecific K2P genetic distances between *H.koatenense* and its congeners range from 1.77% to 2.51% (Table 3). Although the K2P genetic distance between the new combination and *H.guixiense* is 1.77%, it is still larger than other interspecific distances of potamid crabs (e.g., 1.47% between *Nanhaipotamonformosanum* (Parisi, 1916) and *Nanhaipotamondongyinense* Shih, Chen & Wang, 2005; 0.93% between *Nanhaipotamonnanriense* Dai, 1997 and *Nanhaipotamonyongchuense* Dai, 1997; cf. Shih et al. 2011; 1.47% between *Minpotamonnasicum* (Dai, Chen, Song, Fan, Lin & Zeng, 1979) and *Minpotamonkityang* Mao & Huang, 2020; cf. Mao and Huang 2020), and the two species occur far apart (~ 90 km) and can be easily distinguished by the morphological characters in G1 and external orbital teeth. Additionally, we note the smallest genetic distance of 0.27% between *H.medium* and *H.yiyangense*. It is nevertheless comparable with some other pairs of potamid species (e.g., 0.19% between *Aparapotamongrahami* (Rathbun, 1931) and *Aparapotamonbinchuanense* Tan, Zhou & Zou, 2021; cf. Tan et al. 2021; 0.38% between *Aparapotamonsimilium* Dai & Chen, 1985 and *Aparapotamonbinchuanense* Tan, Zhou & Zou, 2021; cf. Tan et al. 2021). Additionally, they can be distinguished from each other by their morphologies (Dai et al. 1995; Cheng et al. 2008). It is noteworthy that the K2P genetic distances between *H.angulatum* and other *Huananpotamon* species are relatively large, ranging from 3.80% to 6.38% (Table 3). However, *H.angulatum* is not morphologically or geographically distinct. Further phylogenetic study of *Huananpotamon* with more extensive sampling of geographical populations, species, and genetic markers is needed. The specimen of *S.fukienense* from the Tongmu Village, the type locality for *S.wuyiensis*, formed a monophyletic group with the specimen from Shaowu City, the type locality for *S.fukienense* (Fig. 9), without distinct genetic difference.

**Table 3.** Genetic distances of K2P pairwise genetic distances based on 16S rDNA sequences among *Huananpotamonkoatenense*, other *Huananpotamon* species, and five taxa from allied genera.

	1	2	3	4	5	6	7	8	9	10	11	12	13	14
1. *Huananpotamonangulatum*														
2. *Huananpotamonchongrenense*	3.80%													
3. *Huananpotamonguixiense*	3.80%	0.92%												
4. *Huananpotamonkoatenense*	4.16%	2.23%	1.77%											
5. *Huananpotamonlichuanense*	5.14%	3.51%	3.09%	2.24%										
6. *Huananpotamonmedium*	5.31%	2.40%	2.19%	2.02%	5.08%									
7. *Huananpotamonsheni*	4.75%	1.34%	0.44%	2.27%	2.75%	0.89%								
8. *Huananpotamonyiyangense*	6.38%	2.56%	1.67%	1.78%	3.18%	0.27%	1.58%							
9. *Huanpotamonlini*	4.49%	1.57%	1.66%	2.51%	3.00%	1.12%	1.66%	0.88%						
10. *Minpotamonnasicum*	9.05%	7.27%	7.03%	5.97%	8.02%	8.84%	7.21%	9.68%	7.21%					
11. *Nanhaipotamonpingyuanense*	8.36%	5.63%	6.12%	5.78%	6.86%	8.92%	7.04%	8.26%	7.04%	9.21%				
12. *Chinapotamondepressum*	8.26%	6.18%	6.44%	6.28%	7.84%	9.14%	7.02%	9.42%	7.62%	7.52%	7.08%			
13. *Qianguimonelongatum*	9.53%	8.87%	8.62%	7.07%	9.97%	11.28%	8.91%	12.06%	9.22%	9.19%	10.08%	7.40%		
14. *Sinolapotamonpatellifer*	7.49%	6.84%	6.62%	6.28%	7.31%	9.05%	7.23%	9.31%	7.23%	7.51%	6.59%	5.89%	8.37%	

## ﻿Discussion

The type specimen of Potamon (Potamon) koatenensis is a female individual whose original type locality is recorded as Koaten (Guadun), Fujian Province, China, inhabiting an altitude of ca 1200 m (Rathbun 1904). The original description of the species is brief and minimally illustrated, and the significance of G1 in freshwater crab classification was not appreciated at that time, making correct judgments based solely on the original description difficult. Follow-up field surveys of the type locality of this species was also lacking. Therefore, it is no wonder that Bott (1970), Ng et al. (2008), and Chu et al. (2018) all followed the classification of Potamon (P.) koatenensis as a member of *Sinopotamon*. Therefore, to confidently resolve the long-standing controversy about the taxonomic status and validity of this species, we conducted comprehensive field surveys and collected specimens of *H.koatenense* in the surrounding areas of Guadun (Koaten) (Fig. 1). As informed by senior villagers, Father Armand David bought specimens from them in a church located in Guadun (Koaten). We therefore speculate that the holotype of *H.koatenense* was probably not actually collected in Guadun. Villagers may have collected the female specimen somewhere nearby, and taken it to the church. Based on the literature and our surveys, a total of eight *Huananpotamon* species are peripatrically distributed in the adjacent regions of the Wuyishan National Park (Fig. 9). To the best of our knowledge, *H.koatenense* is the only *Huananpotamon* species distributed inside and endemic to the Wuyishan National Park.

Previously, there were three freshwater crab species, *S.koatenense*, *S.fukienense*, and *S.wuyiensis* recorded in the area of the park. Based on our comprehensive surveys and research of the crabs in this area, there are still three species, namely, *H.koatenense*, *S.fukienense*, and *B.engelhardti*. The present study sheds some light on the biodiversity of freshwater crabs in the Wuyishan National Park, Fujian Province, China.

## Supplementary Material

XML Treatment for Sinopotamon fukienense

XML Treatment for Huananpotamon koatenense
